# Effectiveness of Transcutaneous Spinal Cord Stimulation for Lower Limb Rehabilitation in Spinal Cord Injury: Protocol for a Systematic Review and Meta-Analysis

**DOI:** 10.2196/80995

**Published:** 2025-10-23

**Authors:** Ravi Shankar, Wei Wen Kevin Sim, Gobinathan Chandran

**Affiliations:** 1 Department of Rehabilitation Medicine, Alexandra Hospital, 378 Alexandra Road, Singapore, 159964, Singapore, 65 83797930; 2 Department of Rehabilitation Medicine, Tan Tock Seng Hospital, Singapore, Singapore; 3 Department of Rehabilitation Medicine, Department of Medicine, National University Hospital, Singapore, Singapore

**Keywords:** spinal cord injury, transcutaneous spinal cord stimulation, tSCS, lower limb rehabilitation, motor function, walking ability, spasticity, neuromodulation

## Abstract

**Background:**

Spinal cord injury (SCI) affects millions of people worldwide and often results in impaired lower limb function and reduced mobility. Transcutaneous spinal cord stimulation (tSCS) has emerged as a promising neuromodulation technique for enhancing motor recovery and walking ability in individuals with SCI.

**Objective:**

This systematic review and meta-analysis will determine the effectiveness of tSCS, compared with sham stimulation, no intervention, or other active interventions, for improving lower limb motor function in individuals with SCI. Secondary objectives are to evaluate tSCS effects on walking ability, spasticity, quality of life, and safety outcomes.

**Methods:**

This systematic review will be conducted according to the Cochrane Handbook for Systematic Reviews of Interventions and reported following the PRISMA (Preferred Reporting Items for Systematic Reviews and Meta-Analyses) 2020 statement. We will search electronic databases (PubMed, Web of Science, Embase, CINAHL, MEDLINE, Cochrane Library, Physiotherapy Evidence Database, and Scopus) from inception to August 2025, along with gray literature, trial registries, and hand-searching of key journals. We will include randomized controlled trials (RCTs), quasi-RCTs, and nonrandomized studies (case series with ≥5 participants) that evaluate the effects of tSCS applied over the spinal region on lower limb rehabilitation outcomes in adults with traumatic or nontraumatic SCI. The primary outcome will be lower limb motor function assessed by validated measures such as the Lower Extremity Motor Score. Secondary outcomes will include walking ability, balance, functional independence, spasticity, quality of life, and adverse events. Two reviewers will independently screen studies, extract data, and assess risk of bias using version 2 of the Cochrane Risk of Bias tool for RCTs and Risk of Bias in Non-randomized Studies of Interventions tool. Data will be synthesized using random-effects meta-analysis if appropriate, with subgroup analyses examining injury level, completeness, stimulation site, and cointervention effects, and sensitivity analyses. Evidence certainty will be evaluated using the Grading of Recommendations Assessment, Development, and Evaluation approach.

**Results:**

This protocol paper presents strategy development and protocol registration, with results expected by October 2025.

**Conclusions:**

This comprehensive systematic review and meta-analysis will address an important evidence gap, providing the first focused evaluation of tSCS effectiveness for lower limb rehabilitation in SCI. Results will inform clinical practice guidelines and guide future research directions.

## Introduction

### Background

Spinal cord injury (SCI) is a devastating condition that affects millions of people worldwide. The global incidence of SCI in 2019 was estimated at 0.9 million new cases [1]. SCI often results from traumatic causes such as road traffic accidents, falls, and violence, but can also be due to nontraumatic etiologies like spinal tumors, infections, and vascular disorders [2]. Depending on the level and severity of the lesion, SCI can lead to a range of impairments, including paralysis, sensory loss, spasticity, pain, autonomic dysfunction, and bowel or bladder dysfunction, which significantly impact functional independence and quality of life [3].

Restoring walking ability is a top priority for many individuals with SCI [4]. Despite significant advancements in rehabilitative therapy, recovery of independent walking remains a major issue for most SCI patients. Reduced physical inactivity and marked deconditioning are highly prevalent among persons with SCI [5]. Physical inactivity is an independent risk factor for cardiovascular disease and premature mortality [6]. Thus, it is imperative to explore various means to increase the physical activity in this group of patients. There is a pressing need for novel interventions that can enhance neuroplasticity and improve functional outcomes in this population.

While various neuromodulation approaches exist for SCI rehabilitation, tSCS represents a unique intervention that specifically targets spinal networks through noninvasive transcutaneous delivery. Unlike broader neuromodulation reviews that encompass diverse techniques with different mechanisms and targets, tSCS requires focused evaluation given its distinct mechanism of activating proprioceptive and cutaneous afferent fibers to modulate spinal cord excitability. The specificity of tSCS for lower limb rehabilitation warrants dedicated examination, as the intervention’s effectiveness may vary significantly based on stimulation site, parameters, and the specific neural pathways involved in lower extremity motor control.

Transcutaneous spinal cord stimulation (tSCS) has emerged as a promising neuromodulation technique for SCI rehabilitation. tSCS involves delivering electrical currents to the spinal cord via surface electrodes placed over the neck or back vertebrae. The stimulation parameters, such as frequency, intensity, and pulse width, can be adjusted to optimize the motor response. The mechanisms of action of tSCS are not fully understood, but computational simulations of cervical tSCS suggest that groups of dorsal root proprioceptive Aα and cutaneous Aβ fibers are coactivated with tSCS at low stimulating intensities [7]. It remains unclear whether similar mechanisms apply to thoracic tSCS protocols. The postulated mechanism by which tSCS affects functional recovery is through the activation of proprioceptive fibers during stimulation, contributing to spinal cord neuromodulation [8,9].

Several studies have investigated the effects of tSCS on lower limb motor function and walking ability in individuals with SCI. Gerasimenko et al [10] first demonstrated that tSCS could enable voluntary leg movements and standing in individuals with motor-complete paraplegia. Subsequent studies have reported improvements in Lower Extremity Motor Scores (LEMS), walking speed, step length, and spasticity with tSCS training in individuals with both complete and incomplete SCI [11-14]. For example, Samejima et al [15] found that a 2-month multisite tSCS program targeting both cervical and lumbosacral regions, combined with intensive locomotor training, increased 6-Minute Walk Test distance threefold compared with locomotor training alone in 2 participants with chronic motor-incomplete tetraplegia. In addition, both participants demonstrated improvements in balance, bowel function, and sensory recovery, with 1 participant eliminating the need for intermittent catheterization following the intervention.

Despite these promising findings, the evidence on tSCS for SCI rehabilitation remains limited by small sample sizes, heterogeneous protocols, and a lack of long-term follow-up data. It is unclear which subgroups of individuals with SCI may benefit most from tSCS, what the optimal stimulation parameters and training regimens are, and whether the effects are maintained over time. A comprehensive synthesis of the current literature is needed to inform clinical practice guidelines and guide future research directions. Recent methodological reviews have highlighted the importance of standardized approaches to tSCS research [16,17], while emerging evidence continues to expand our understanding of optimal stimulation protocols and patient selection criteria.

To date, there has been no systematic review specifically focused on evaluating the effectiveness of tSCS for lower limb rehabilitation in people with SCI. While several reviews have examined neuromodulation techniques in SCI, they have either been broader in scope or focused on different aspects. García et al [18] analyzed tSCS across 13 studies but covered both upper and lower extremities, without a specific focus on lower limb outcomes. Most recently, García-Alén et al [19] performed a systematic review and meta-analysis specifically examining noninvasive electromagnetic neuromodulation for upper limb motor strength and functionality in cervical SCI, further highlighting the gap in systematic evaluation of tSCS specifically for lower limb rehabilitation. Barss et al [20] examined tSCS effects on lumbar networks, but this work focused on physiological mechanisms rather than providing a comprehensive literature review of rehabilitation outcomes.

Most recently, Hernandez-Navarro et al [21] conducted a systematic review examining both noninvasive brain and spinal cord stimulation for motor and gait recovery in incomplete SCI. Their review was restricted to randomized controlled trials (RCTs) scoring above 5 on the Physiotherapy Evidence Database (PEDro) scale and only included 1 tSCS study [22], indicating a need for more comprehensive evaluation of tSCS evidence. Our systematic review addresses this specific gap by focusing exclusively on tSCS, using broader inclusion criteria for study designs (including nonrandomized studies and case series with ≥5 participants), and analyzing a wider range of clinical outcomes including spasticity and quality of life. This approach provides a more comprehensive assessment of the emerging evidence for tSCS in SCI rehabilitation by capturing studies that were excluded from previous reviews due to methodological constraints. In addition, our review will include newer studies published since the search period for Hernandez-Navarro et al [21] ended in March 2024, incorporating the latest evidence until March 2025.

Furthermore, our systematic review methodology uses a more comprehensive search strategy across multiple databases including PubMed, Web of Science, Embase, CINAHL, Cochrane Library, PEDro, and Scopus, compared with the 3 databases used by Hernandez-Navarro et al [21]. Our review extends the scope of outcome measures to include important functional outcomes, such as Spinal Cord Independence Measure and quality of life assessments (EuroQol-5 Dimension [EQ-5D]), providing a more holistic evaluation that addresses meaningful patient-centered outcomes. Our planned subgroup analyses examining injury type, stimulation parameters, and stimulation site will allow for more nuanced conclusions about optimal tSCS protocols for different SCI populations. The GRADE (Grading of Recommendations Assessment, Development and Evaluation) approach for evidence evaluation will provide clearer clinical guidance, while our inclusion of safety and adverse event reporting will address an important gap in understanding the risk-benefit profile of tSCS interventions across different SCI populations.

Given the limitations of previous reviews and the growing body of research on tSCS for SCI, a comprehensive and up-to-date systematic review with meta-analysis is warranted. This will help to determine the overall efficacy and safety of tSCS as a lower limb rehabilitation strategy for SCI, identify factors that may influence treatment response, and guide the design of future clinical trials.

### Objectives

This systematic review and meta-analysis aims to determine the effectiveness of tSCS compared with sham stimulation, no intervention, or other active interventions for improving lower limb motor function, as measured by validated assessments such as the LEMS, in individuals with SCI. Secondary objectives include evaluating tSCS effects on walking ability (speed, distance, and independence), spasticity, quality of life, and safety outcomes. The review will also examine potential treatment effect moderators, including injury characteristics (level, severity, and chronicity), stimulation parameters (frequency, intensity, and site), and co-interventions to guide optimal tSCS implementation protocols.

## Methods

### Overview

This systematic review will be conducted in accordance with the Cochrane Handbook for Systematic Reviews of Interventions [23] and reported following the PRISMA (Preferred Reporting Items for Systematic Reviews and Meta-Analyses) 2020 statement [24]. Any amendments to this registered protocol will be documented and reported transparently, including the date, rationale, and description of changes. Major amendments that could affect study conclusions will be highlighted in the final review publication to ensure protocol transparency and adherence monitoring. The PRISMA flow diagram will visually summarize the study selection process, including the number of records identified, included, and excluded, and the reasons for exclusions at each stage.

This systematic review is in the planning and protocol development phase. The search strategy has been developed and the protocol has been registered with PROSPERO, but formal screening of records has not yet begun. We anticipate completing the record screening by June 2025, followed by data extraction by August 2025. Analysis, synthesis, and results are expected to be available by October 2025. We confirm that our systematic review is prospective in nature, with data collection and analysis planned but not yet executed. This protocol describes the methodology that will be used to conduct the review once all preliminary steps are completed.

The Population, Intervention, Comparator, and Outcome (PICO) framework was used to design this systematic review protocol. The PICO framework is a widely used tool for formulating research questions and eligibility criteria in systematic reviews of interventions [25]. It helps to ensure that the review is focused, clinically relevant, and captures all key aspects of the topic.

In this protocol, the PICO elements were defined as (1) population: adults with SCI of any level, severity, or chronicity; (2) intervention: active tSCS; (3) comparator: sham tSCS, no intervention, conventional rehabilitation, or other active interventions; and (4) outcomes: primary—lower limb motor function, and secondary—walking ability, spasticity, quality of life, and adverse events.

Using the PICO framework, we developed a clearly defined review question: “What is the effectiveness of tSCS compared with sham stimulation, no intervention, or other active interventions for improving lower limb motor function, walking ability, spasticity, and quality of life in adults with spinal cord injury?” This question guided the selection of eligibility criteria, search terms, and data extraction variables.

### Eligibility Criteria

#### Study Designs

We will include RCTs, quasi-RCTs, and nonrandomized studies (cohort studies, case-control studies, and case series with ≥5 participants) that evaluate the effects of tSCS on lower limb rehabilitation outcomes in individuals with SCI. To manage the anticipated heterogeneity from diverse study designs, we will implement a hierarchical synthesis approach. RCTs will be analyzed separately from nonrandomized studies, with primary conclusions drawn from RCT evidence where available. Nonrandomized studies will be used to supplement RCT findings and explore treatment effects in populations or contexts not adequately represented in controlled trials. Meta-analysis will only be attempted when studies demonstrate sufficient clinical and methodological homogeneity, with narrative synthesis used when heterogeneity precludes quantitative pooling. Crossover trials will be included if data from the first treatment period can be obtained. Conference abstracts, case reports, qualitative studies, narrative reviews, editorials, and expert opinions will be excluded.

To address concerns about methodological heterogeneity from including diverse study designs, we have established strict criteria for synthesis. Only studies with sufficient clinical and methodological homogeneity will be included in meta-analyses, with a minimum threshold of *I*² <75% for pooling. When heterogeneity exceeds this threshold, we will conduct narrative synthesis only. Furthermore, we will weight evidence hierarchically, with RCTs forming the primary evidence base and nonrandomized studies serving as supplementary evidence to explore treatment effects in populations or contexts not represented in controlled trials. Case series will be analyzed separately and used primarily to assess safety outcomes and identify potential treatment responders, rather than contributing to efficacy estimates.

#### Participants

Studies involving adults (≥18 y old) with traumatic or nontraumatic SCI, regardless of level of injury, severity (American Spinal Injury Association Impairment Scale [AIS] grades A-D), or chronicity (acute, subacute, and chronic) of injury will be included. Studies with mixed populations (eg, SCI and stroke) will be included only if SCI-specific data can be extracted. Studies involving children (<18 y old) or individuals with other neurological conditions without SCI will be excluded.

#### Interventions

The intervention of interest is tSCS applied over the neck or back using surface electrodes, with or without simultaneous physical training, for the purpose of improving lower limb motor function and walking ability. Studies must report the stimulation parameters used, including frequency, intensity, pulse width, and duration of application. tSCS can be delivered alone or in combination with other interventions. Studies using other types of electrical stimulation (eg, functional electrical stimulation and epidural stimulation) without tSCS will be excluded.

#### Comparators

Eligible comparators include (1) sham tSCS (using identical procedures but with no actual stimulation), (2) no intervention, (3) conventional rehabilitation (eg, physical therapy and overground walking training), and (4) other active interventions (eg, robotic gait training, exoskeletons, and bodyweight-supported treadmill training).

Studies comparing different tSCS parameters or combined interventions will also be included. We will not pool data from different comparator groups (sham stimulation, no intervention, and other active interventions) in our primary analyses. Instead, we will conduct separate meta-analyses for each comparison type and perform subgroup analyses to examine differential effects across comparator categories. This approach will preserve the distinct mechanisms and expected effect sizes associated with different control conditions.

### Outcomes

The primary outcome will be lower limb motor function, as measured by a validated assessment tool such as the LEMS [26] and International Standards for Neurological Classification of Spinal Cord Injury (ISNCSCI) [27]. The LEMS represents a subset of the total ISNCSCI motor score, focusing specifically on lower extremity muscle groups. When studies report only total ISNCSCI motor scores, we will contact authors to obtain LEMS-specific data. If multiple measures are reported, LEMS will be prioritized as it is the most widely used outcome in SCI clinical trials. To address the breadth of outcomes, we will establish a clear hierarchy for interpretation and clinical recommendations. The primary outcome (LEMS) will be the main focus for drawing conclusions about tSCS effectiveness. For secondary outcomes, we will prioritize patient-important functional outcomes in the following order: (1) walking independence (Walking Index for Spinal Cord Injury), (2) walking speed and distance, (3) functional independence (Spinal Cord Independence Measure), and (4) quality of life measures. Motor impairment measures will be considered surrogate outcomes, with clinical recommendations based primarily on functional outcomes when both are available. This prioritization will be applied consistently in the results synthesis and clinical interpretation.

Secondary outcomes will include (1) walking speed (eg, 10-Meter Walk Test), (2) walking distance (eg, 6-min Walk Test), (3) walking independence (eg, Walking Index for Spinal Cord Injury), (4) spasticity (eg, Modified Ashworth Scale), (5) balance (eg, Berg Balance Scale), (6) functional independence (eg, Spinal Cord Independence Measure), (7) quality of life (eg, EQ-5D and Spinal Cord Injury Quality of Life [SCI-QoL]; we will consider including the SCI-QoL measure in addition to or instead of the EQ-5D, given concerns about the relevance and fairness of EQ-5D questions for the SCI population, and (8) adverse events (eg, pain, skin irritation, and autonomic dysreflexia).

Outcome measures at all time points (eg, preintervention, postintervention, and follow-up) will be extracted. For studies with multiple follow-up assessments, we will prioritize consistency in timepoints across studies for meta-analysis. The selection of follow-up time points will be determined after data collection to optimize homogeneity across included studies.

To clearly define the scope of this systematic review, the inclusion and exclusion criteria are summarized in Table 1 below:

**Table 1.** Inclusion and exclusion criteria.

Variables	Inclusion criteria	Exclusion criteria
Population	Adults (≥18 y old) with traumatic or nontraumatic spinal cord injury of any level, severity, or chronicity	Adults aged <18 y old; neurological conditions other than spinal cord injury
Interventions	tSCS^a^ alone or combined with other interventions	Other types of electrical stimulation without tSCS (eg, functional electrical stimulation and epidural stimulation)
Comparators	Sham tSCS, no intervention, conventional rehabilitation, or other active interventions	None
Outcomes	Primary: lower limb motor function; and secondary: walking ability, spasticity, quality of life, and adverse events	Nonmotor outcomes (eg, cardiorespiratory function, pain, and bowel or bladder function)
Study designs	RCTs^b^, quasi-RCTs, cohort studies, case-control studies, case series (≥5 participants)	Case reports, cross-sectional studies, qualitative studies, reviews, editorials, commentaries, animal studies

a tSCS: transcutaneous spinal cord stimulation.

b RCT: randomized controlled trial.

### Information Sources and Search Strategy

We will conduct a comprehensive literature search of the following electronic databases from their inception to August 2025: PubMed (MEDLINE), Web of Science, Embase, CINAHL, The Cochrane Library (CENTRAL), PEDro, and Scopus. The search strategy will be developed in consultation with experts and include a combination of keywords and controlled vocabulary terms (eg, Medical Subject Headings) related to SCI, tSCS, and lower limb rehabilitation outcomes. No language or publication date filters will be applied. The search will be adapted for each database and peer-reviewed using the Peer Review of Electronic Search Strategies checklist [28].

A draft search strategy for PubMed is provided below:

((spinal cord injur*[Title/Abstract] OR SCI[Title/Abstract] OR paraplegi*[Title/Abstract] OR quadriplegi*[Title/Abstract] OR tetraplegi*[Title/Abstract]) AND (transcutaneous spinal cord stimulat*[Title/Abstract] OR trans-cutaneous spinal cord stimulat*[Title/Abstract] OR tSCS[Title/Abstract] OR “transcutaneous electrical spinal stimulation”[Title/Abstract] OR “non-invasive spinal cord stimulation”[Title/Abstract] OR “noninvasive spinal cord stimulation”[Title/Abstract]) AND (lower limb[Title/Abstract] OR lower extremit*[Title/Abstract] OR leg[Title/Abstract] OR gait[Title/Abstract] OR locomotion[Title/Abstract] OR walk*[Title/Abstract] OR motor function*[Title/Abstract]))

In addition to electronic databases, we will search the following sources to identify potentially relevant studies:

- ClinicalTrials website
- World Health Organization International Clinical Trials Registry Platform
- OpenGrey (for gray literature)
- Reference lists of included studies and pertinent reviews
- Hand-search of key journals (eg, Spinal Cord, Journal of Neurotrauma, and Journal of Neuroengineering and Rehabilitation)

We will also contact experts in the field, including authors of included studies, to inquire about ongoing or unpublished trials.

### Study Selection

The results of the literature search will be uploaded to Covidence, a web-based systematic review management platform. After removal of duplicates, 2 reviewers will independently screen titles and abstracts against the eligibility criteria. Studies that clearly do not meet the inclusion criteria will be excluded. The full-text articles of the remaining studies will be retrieved and assessed for eligibility by the 2 reviewers independently. Any disagreements will be resolved through discussion or consultation with a third reviewer. Reasons for exclusion at the full-text stage will be recorded and reported in a PRISMA flow diagram. The study selection process will be pilot-tested on a sample of articles to ensure consistency between reviewers.

### Data Extraction

Data from included studies will be extracted independently by 2 reviewers using a standardized form in Covidence. The form will be piloted on a subset of studies and refined as necessary. Disagreements will be resolved through consensus or adjudication by a third reviewer. Two reviewers will independently extract data using a comprehensive framework that captures multiple domains of information. This systematic approach will ensure thorough data collection across all included studies while maintaining consistency and reliability in the extraction process.

A detailed data extraction framework has been developed to capture key information from each study, encompassing study characteristics, methodological approaches, participant demographics, intervention specifics, comparison groups, outcome measurements, and study quality indicators. This structured approach ensures standardized data collection that will facilitate subsequent analysis and synthesis of the evidence.

The complete data extraction framework is detailed in Multimedia Appendix 1, which provides a comprehensive outline of all data fields to be extracted. These include study details (authorship, publication information, and funding), methodological aspects (study design, setting, and recruitment), participant characteristics (demographic and clinical data), intervention and comparator specifications (parameters, protocols, and schedules), outcome measures (primary and secondary end points, measurement tools, and time points), and study limitations (potential biases, data gaps, and methodological concerns). This detailed extraction protocol will enable robust analysis and synthesis of the evidence while maintaining consistency between reviewers.

If data are missing or unclear, we will attempt to contact the study authors for clarification. Studies will be excluded if key data cannot be obtained after 3 attempts. We will also contact authors of included studies to request access to individual participant data when published aggregate data are insufficient for planned subgroup analyses. This approach will enable more detailed examination of treatment effects across different injury characteristics and stimulation parameters.

### Risk of Bias Assessment

Risk of bias in individual studies will be assessed independently by 2 reviewers using version 2 of the Cochrane Risk of Bias tool (RoB 2) [29] for RCTs and the Risk of Bias in Non-randomized Studies of Interventions (ROBINS-I) tool [30] for nonrandomized studies. RoB 2 evaluates 5 domains of bias: randomization process, deviations from intended interventions, missing outcome data, measurement of the outcome, and selection of the reported result. Each domain is judged as low risk, some concerns, or high risk. ROBINS-I assesses 7 domains: confounding, selection of participants, classification of interventions, deviations from intended interventions, missing data, measurement of outcomes, and selection of reported results. The overall risk of bias is categorized as low, moderate, serious, or critical.

Disagreements between reviewers will be resolved through discussion or referral to a third reviewer. The risk of bias judgments for each study will be presented in a “traffic light” plot and summarized in a risk of bias table. The results will be used to guide the interpretation of the review findings and assess the certainty of the evidence.

### Data Synthesis

If 2 or more studies report the same outcome, we will conduct a meta-analysis using a random-effects model in RevMan 5.4 software (Cochrane). Continuous outcomes will be pooled using mean differences or standardized mean differences with 95% CIs, depending on whether the same or different measurement scales are used across studies. Dichotomous outcomes will be combined using risk ratios with 95% CI. If data are missing or cannot be converted to the required format, we will use alternative methods such as the generic inverse variance or contact study authors for additional information.

Statistical heterogeneity will be assessed using the *I*^2^ statistic and chi-square test. *I*^2^ values of 25%, 50%, and 75% represent low, moderate, and high heterogeneity, respectively [31]. If substantial heterogeneity is present (*I*^2^>50% or *P*<.10), we will explore possible sources through subgroup and sensitivity analyses. If a meta-analysis is not appropriate due to clinical or methodological heterogeneity, we will provide a narrative synthesis of the findings, including tables and figures to aid in data presentation.

### Subgroup and Sensitivity Analyses

If sufficient data are available, we will perform subgroup analyses to investigate potential effect modifiers and sources of heterogeneity. The following subgroups will be considered:

- Level of injury: cervical versus thoracic versus lumbosacral
- Severity of injury: complete (AIS A) versus incomplete (AIS B-D)
- Type of injury: traumatic versus atraumatic
- Time since injury: acute (<1 mo) versus subacute (1‐6 mo) versus chronic (>6 mo)
- Site of stimulation: cervical versus thoracic versus lumbosacral
- tSCS frequency: low (<50 Hz) versus high (≥50 Hz) tSCS waveform: monophasic versus biphasic versus both tSCS intensity: different intensity parameters as defined in individual studies
- Cointerventions: tSCS alone versus tSCS+physical or functional training
- Carrier frequency: studies using kilohertz (kHz) carrier frequencies versus conventional frequencies, given ongoing debate in the field regarding optimal stimulation parameters

We will use the test for subgroup differences in RevMan to determine whether the effect of tSCS differs significantly between subgroups.

Given the anticipated limited number of studies, we will prioritize subgroup analyses based on clinical importance and statistical feasibility. We will only conduct subgroup analyses when there are at least 3 studies per subgroup and a minimum of 10 total studies for the outcome. The primary subgroup analyses will focus on injury completeness (AIS A vs B-D) and cointerventions (tSCS alone vs combined), as these are most likely to be clinically meaningful and adequately powered. Secondary subgroup analyses will only be performed if sufficient studies are available, and results will be interpreted cautiously with clear acknowledgment of limited power.

Sensitivity analyses will be conducted to assess the robustness of the meta-analysis results. We will repeat the analysis excluding studies at high risk of bias, studies with missing data, and outliers (studies with effect estimates that are substantially different from the pooled estimate). If the results remain largely unchanged, we will consider the findings to be stable. If the results change substantially, we will explore potential reasons and interpret the results with caution.

### Meta-Biases

To assess publication bias, we will create funnel plots for each outcome if there are at least 10 studies in the meta-analysis. Asymmetry in the funnel plot may indicate the presence of publication bias or small-study effects. We will also use the test by Egger et al [32] to quantitatively assess funnel plot asymmetry. If evidence of publication bias is found, we will use the trim-and-fill method [33] to estimate the impact on the pooled effect estimate.

### Confidence in Cumulative Evidence

The strength of the body of evidence for each outcome will be assessed using the GRADE approach [34]. In interpreting results, patient-important functional outcomes (walking independence and quality of life measures) will be prioritized over surrogate motor assessments when drawing clinical conclusions. Evidence from RCTs will be weighted more heavily than nonrandomized studies, with separate synthesis and interpretation strategies used for different evidence levels to prevent dilution of high-quality findings. Two reviewers will independently evaluate the evidence for limitations in study design, inconsistency, indirectness, imprecision, and publication bias. The quality of evidence will be rated as high, moderate, low, or very low for each outcome. Disagreements will be resolved through discussion or input from a third reviewer. The GRADE assessments will be presented in a summary of findings table.

## Results

**Figure 1.**
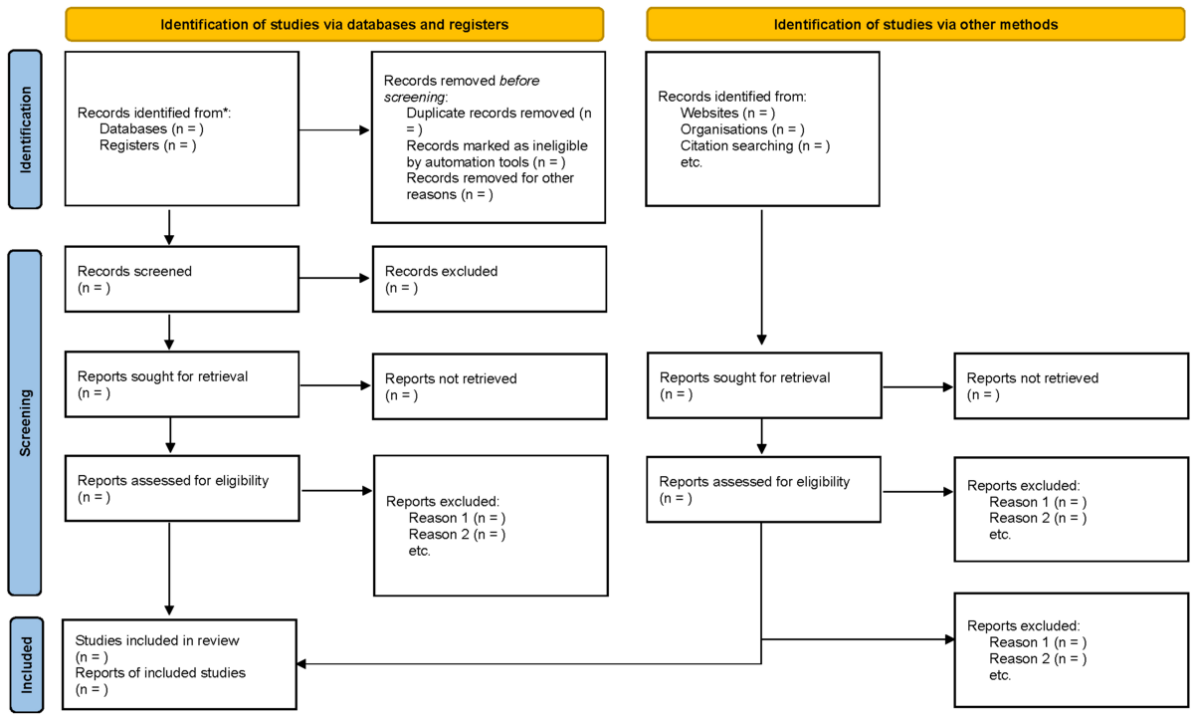
PRISMA (Preferred Reporting Items for Systematic Reviews and Meta-Analyses) flow diagram.

The study selection process will be documented using a PRISMA flow diagram, which will detail the number of records identified, screened, excluded, and included at each stage of the review process (Figure 1). This systematic review protocol was funded and registered with PROSPERO in February 2025. The comprehensive search strategy has been finalized and peer-reviewed. Systematic database searching is scheduled to begin in March 2025, followed by title and abstract screening through May 2025. Full-text review and data extraction are projected for June-August 2025, with meta-analysis and manuscript preparation planned for September-October 2025. Results publication is expected in the first quarter of 2026. No recruitment is applicable as this is a systematic review of published literature.

## Discussion

### Anticipated Principal Findings

This systematic review is expected to reveal heterogeneous effects of tSCS across different SCI populations and stimulation protocols. Based on existing literature suggesting variable responses to neuromodulation interventions, we anticipate finding that individuals with incomplete injuries (AIS B-D) will demonstrate significantly greater improvements in lower limb motor function compared with those with complete injuries (AIS A). We expect effect sizes to be moderate for incomplete injuries (standardized mean difference 0.5‐0.8) and minimal for complete injuries. In addition, we anticipate that combined tSCS and physical training protocols will show superior outcomes compared with tSCS alone, with combined interventions potentially doubling the treatment effect size, supporting the concept of activity-dependent neuroplasticity.

### Comparison With Previous Work

This review will provide the first comprehensive meta-analytic synthesis specifically focused on tSCS for lower limb rehabilitation in SCI, addressing a critical gap identified in previous broader reviews. Unlike the review by Comino-Suárez et al [35], which covered both upper and lower extremities without a specific focus, and Hernandez-Navarro et al [21] which had a restrictive inclusion of only high-quality RCTs, our approach balances methodological rigor with the practical reality of the emerging tSCS evidence base. Our findings will either support or challenge preliminary conclusions from individual studies and smaller reviews, potentially revealing whether early promising results from case series translate to meaningful effects in controlled trials.

### Methodological Strengths and Limitations

This protocol incorporates several methodological strengths including prospective PROSPERO registration, comprehensive search across 8 databases, and rigorous quality assessment using validated tools (RoB 2 and ROBINS-I). The hierarchical synthesis approach acknowledges the current research landscape while maintaining scientific rigor. However, important limitations must be acknowledged. The inclusion of diverse study designs, while comprehensive, may introduce substantial heterogeneity that could limit meta-analysis feasibility. The emerging nature of tSCS research suggests many included studies may have small sample sizes and limited follow-up, potentially affecting evidence quality and our ability to detect clinically meaningful effects. The broad range of outcome measures, while providing comprehensive coverage, may dilute focus and complicate identification of the most clinically relevant treatment effects.

### Clinical Implications and Future Directions

The results will directly inform clinical decision-making by identifying optimal tSCS protocols, appropriate patient populations, and realistic expectations for functional improvement. If our hypotheses are confirmed, clinical guidelines should prioritize tSCS for individuals with incomplete SCI and emphasize combined stimulation-training protocols. The review will also identify critical research gaps, potentially revealing needs for standardized outcome measures, longer follow-up studies, and adequately powered trials in specific populations. Future research should focus on dose-response relationships, maintenance protocols, and cost-effectiveness analyses to support health care implementation decisions.

### Dissemination Plan

Results will be disseminated through multiple channels to maximize clinical impact. Primary dissemination will include publication in a high-impact rehabilitation or neurology journal, presentation at major SCI conferences (International Spinal Cord Society and American Congress of Rehabilitation Medicine), and development of clinical practice recommendations. We will create accessible summaries for patient organizations and rehabilitation professionals and work with clinical guideline developers to incorporate evidence-based recommendations. Social media and professional networks will be leveraged to reach practicing clinicians globally, ensuring research findings translate to improved patient care.

### Conclusions

This systematic review protocol establishes a methodologically rigorous framework for evaluating tSCS effectiveness in SCI rehabilitation while acknowledging the challenges inherent in synthesizing evidence from an emerging intervention field. The hierarchical synthesis approach and outcome prioritization strategy will ensure that clinical recommendations are based on the highest quality evidence available while capturing the full scope of current research. Anticipated findings of heterogeneous treatment effects across injury types and stimulation protocols will provide crucial guidance for clinical implementation and future research priorities. By October 2025, this review will deliver evidence-based recommendations for tSCS use in SCI rehabilitation, informing both clinical practice guidelines and research funding priorities as this promising neuromodulation technique continues to evolve.

## Supplementary material

10.2196/80995 Multimedia Appendix 1 Data extraction form.

## Abbreviations

AIS: Association Impairment Scale
EQ-5D: EuroQol-5 Dimension
GRADE: Grading of Recommendations Assessment, Development and Evaluation
ISNCSCI: International Standards for Neurological Classification of Spinal Cord Injury
LEMS: Lower Extremity Motor Score
PEDro: Physiotherapy Evidence Database
PICO: Population, Intervention, Comparator, and Outcome
PRISMA: Preferred Reporting Items for Systematic Reviews and Meta-Analyses
RCT: randomized controlled trial
RoB 2: version 2 of the Cochrane Risk of Bias
ROBINS-I: Risk of Bias in Non-randomized Studies of Interventions
SCI: spinal cord injury
SCI-QoL: Spinal Cord Injury Quality of Life
tSCS: transcutaneous spinal cord stimulation
