# Supplementary material for: Effectiveness of Transcutaneous Spinal Cord Stimulation for Lower Limb Rehabilitation in Spinal Cord Injury: Protocol for a Systematic Review and Meta-Analysis
Source: JMIR Res Protoc. 2025 Oct 23;14:e80995. doi: 10.2196/80995 (PMC12548825; doi:10.2196/80995)
Supplement: Multimedia Appendix 1 [file resprot-v14-e80995-s001.docx]

| **S.No** | **Category** | **Variable** |
| --- | --- | --- |
|  | Study Characteristics | First author |
|  |  | Year of publication |
|  |  | Country |
|  |  | Funding source |
|  |  | Study design (e.g. RCT, cohort study) |
|  |  | Setting (e.g. inpatient, outpatient) |
|  |  | Recruitment method (e.g. consecutive, convenience) |
|  |  | Sample size (total and per group) |
|  |  | Inclusion criteria |
|  |  | Exclusion criteria |
|  | Participant Characteristics | Age (mean, SD, range) |
|  |  | Sex (male/female) |
|  |  | Level of injury (cervical/thoracic/lumbar-sacral) |
|  |  | Severity of injury (complete/incomplete, AIS grade) |
|  |  | Time since injury (mean, SD, range) |
|  |  | Ambulatory status (non-ambulatory/ambulatory) |
|  |  | Comorbidities |
|  |  | Medications |
|  | Intervention Details | tSCS device (manufacturer, model) |
|  |  | Stimulation parameters (frequency, intensity, pulse width) |
|  |  | Electrode placement (vertebral level, paramedian/midline) |
|  |  | Session duration and frequency |
|  |  | Total number of sessions |
|  |  | Co-interventions (type, dose) |
|  |  | Treatment adherence/compliance |
|  | Comparator Details | Type (sham tSCS, no treatment, active intervention) |
|  |  | Sham method (e.g. minimal intensity) |
|  |  | Active comparator parameters (type, dose) |
|  |  | Session duration and frequency |
|  |  | Total number of sessions |
|  | Outcome Measures | Lower limb motor function - Measure used (e.g. LEMS, ISNCSCI) |
|  |  | Lower limb motor function - Scores at baseline and follow-up |
|  |  | Lower limb motor function - Change from baseline |
|  |  | Lower limb motor function - Between-group difference |
|  |  | Lower limb motor function - Effect size |
|  |  | Walking ability - Measures used (e.g. 10MWT, 6MWT, FIM-L) |
|  |  | Walking ability - Scores at baseline and follow-up |
|  |  | Walking ability - Change from baseline |
|  |  | Walking ability - Between-group difference |
|  |  | Walking ability - Effect size |
|  |  | Spasticity - Measure used (e.g. MAS) |
|  |  | Spasticity - Scores at baseline and follow-up |
|  |  | Spasticity - Change from baseline |
|  |  | Spasticity - Between-group difference |
|  |  | Spasticity - Effect size |
|  |  | Quality of life - Measure used (e.g. SF-36) |
|  |  | Quality of life - Scores at baseline and follow-up |
|  |  | Quality of life - Change from baseline |
|  |  | Quality of life - Between-group difference |
|  |  | Quality of life - Effect size |
|  |  | Adverse events - Type (e.g. pain, skin irritation) |
|  |  | Adverse events - Number of events |
|  |  | Adverse events - Severity (mild/moderate/severe) |
|  |  | Adverse events - Causality (related/unrelated) |
|  | Study Limitations | Sources of bias (selection, performance, detection, attrition, reporting, other) |
|  |  | Missing data |
|  |  | Methodological issues |
|  |  | Generalizability |
|  |  | Conflicts of interest |
